# Chloroplast development at low temperature requires the pseudouridine synthase gene *TCD3* in rice

**DOI:** 10.1038/s41598-020-65467-2

**Published:** 2020-05-22

**Authors:** Dongzhi Lin, Rongrong Kong, Lu Chen, Yulu Wang, Lanlan Wu, Jianlong Xu, Zhongze Piao, Gangseob Lee, Yanjun Dong

**Affiliations:** 1 College of Life Sciences, Shanghai Normal University, Shanghai, 200234 China; 2 The Institute of Crop Sciences, Chinese Academy of Agricultural Sciences, 12 South Zhong-Guan Cun Street, Beijing, 100081 China; 3 Crop Breeding and Cultivation Research Institute, Shanghai Academy of Agricultural Sciences, 1000 Jingqi Road, Fengxian District Shanghai, 201403 China; 4 National Institute of Agricultural Science, Jeon Ju, 560-500 Korea; 5 Shanghai Key Laboratory of Plant Molecular Sciences, Shanghai, 200234 China

**Keywords:** Behavioural genetics, Behavioural genetics, Plant genetics, Plant molecular biology, Plant molecular biology

## Abstract

Low temperature affects a broad spectrum of cellular components in plants, such as chloroplasts, as well as plant metabolism. On the other hand, pseudouridine (Ψ) synthases are required for the most abundant post-transcriptional modification of RNA in *Escherichia coli*. However, the role of rice Ψ synthases in regulating chloroplast development at low temperature remains elusive. In this study, we identified the rice thermo-sensitive chlorophyll-deficient (*tcd3*) mutant, which displays an albino phenotype before the 4-leaf stage and ultimately dies when grown at 20 °C, but can grow normally at 32 °C. Genetic analysis showed that the mutant trait is controlled by a single recessive nuclear gene (*tcd3*). Map-based cloning, complementation and knockout tests revealed that *TCD3* encodes a chloroplast-localized Ψ synthase. *TCD3* is a cold-induced gene that is mainly expressed in leaves. The disruption of *TCD3* severely affected the transcript levels of various chloroplast-associated genes, as well as ribosomal genes involved in chloroplast rRNA assembly at low temperature (20 °C), whereas the transcript levels of these genes were normal at high temperature (32 °C). These results provide a first glimpse into the importance of rice Ψ synthase gene in chloroplast development at low temperatures.

## Introduction

Rice is one of the most important food crops worldwide. Its yield potential is limited by the photosynthetic capacity of leaves that, as carbohy-drate factories, are unable to fill the larger number of florets of modern rice plants^1^. The development of intact chloroplasts, a prerequisite for photosynthesis, is affected by environmental factors such as temperature and light. Chloroplast development, a complex process that is coordinately regulated by plastid and nuclear genes, can be divided into three stages^2–4^: (i) the activation of plastid replication and plastid DNA synthesis; (ii) chloroplast “build-up”, characterized by the establishment of the chloroplast genetic system; and (iii) the expression of plastid and nuclear genes for the photosynthetic apparatus. In higher plants, chloroplast genes are transcribed by two types of RNA polymerase: nucleus-encoded polymerases (NEP) and plastid-encoded polymerases (PEP). NEPs are mainly responsible for transcribing the components of the transcriptional/translational machinery, such as *rpoA* and *rpoB*, while PEPs are required for the transcription of photosynthetic genes such as *psbA*, *psbD*, and *rbcL*. Mutations of those genes directly or indirectly affect chlorophyll biosynthesis or degradation pathways as well as photosynthesis, ultimately resulting in differences in leaf color or even plant death^5–9^. However, the roles of the many genes in the regulation of chloroplast development in higher plants remain largely unknown^10^.

Pseudouridine (5-ribosyluracil; Ψ) synthases, responsible for the most abundant post-transcriptional modification of cellular RNAs (pseudouridine), share a common core fold and active site structure. This core structure is modified by peripheral domains comprising accessory proteins with different amino acid sequences (depending on the family) and guide RNAs, giving rise to remarkable substrate versatility. These synthases catalyze the site-specific isomerization of uridine residues in the RNA chain and appear to employ both sequence and structural information to achieve site specificity^11^. All Ψ synthases identified to date from Archaea, Bacteria, and Eukarya can be classified into five families (RluA, RsuA, TruA, TruB, and TruD)^12^. The most well studied are from *Escherichia coli*, which contains 11 Ψ synthases. Of these, RsuA, RluE, RluB, and RluF belong to the RsuA family; RluA, RluC, RluD, and TruC belong to the RluA family; and TruA, TruB, and TruD are the sole members of their respective families. All of these synthases are site specific, and no overlapping functions have been detected^12,13^. For instance, RsuA isomerizes U516 in ribosomal RNA (rRNA), which is responsible for the universally conserved Ψ55 in the TΨC loops of all elongator tRNAs in a cell. RluA modifies two structurally distinct types of RNA at positions that share local sequence and structural similarity. TruA and RluD modify several nearby sites on a specific RNA^11^. Nevertheless, the modes of action of Ψ synthases remain largely unclear, especially during plant growth and development. *Chlamydomonas reinhardtii Maa2*, encoding a Ψ synthases, is involved in chloroplast group II intron trans-splicing of *psaA* RNA; its mutant (*maa2*) cannot grow phototrophically and is highly photosensitive^14^. The *Arabidopsis thaliana* Ψ synthase SVR1 (At2g39140) is required for normal chloroplast translation^15^. However, to our knowledge, no rice mutants for Ψ synthase genes have been reported, and the roles of these genes in rice are unknown.

Here, we identified *tcd3*, a mutant of a pseudouridine (Ψ) synthase gene in rice that displays thermo-sensitive changes in leaf color under cold stress. The chloroplast-localized Ψ synthase TCD3 appears to play an essential role in chloroplast development at low temperature in rice.

## Results

### Phenotypic characterization of the *tcd3* mutant

**Figure 1:**
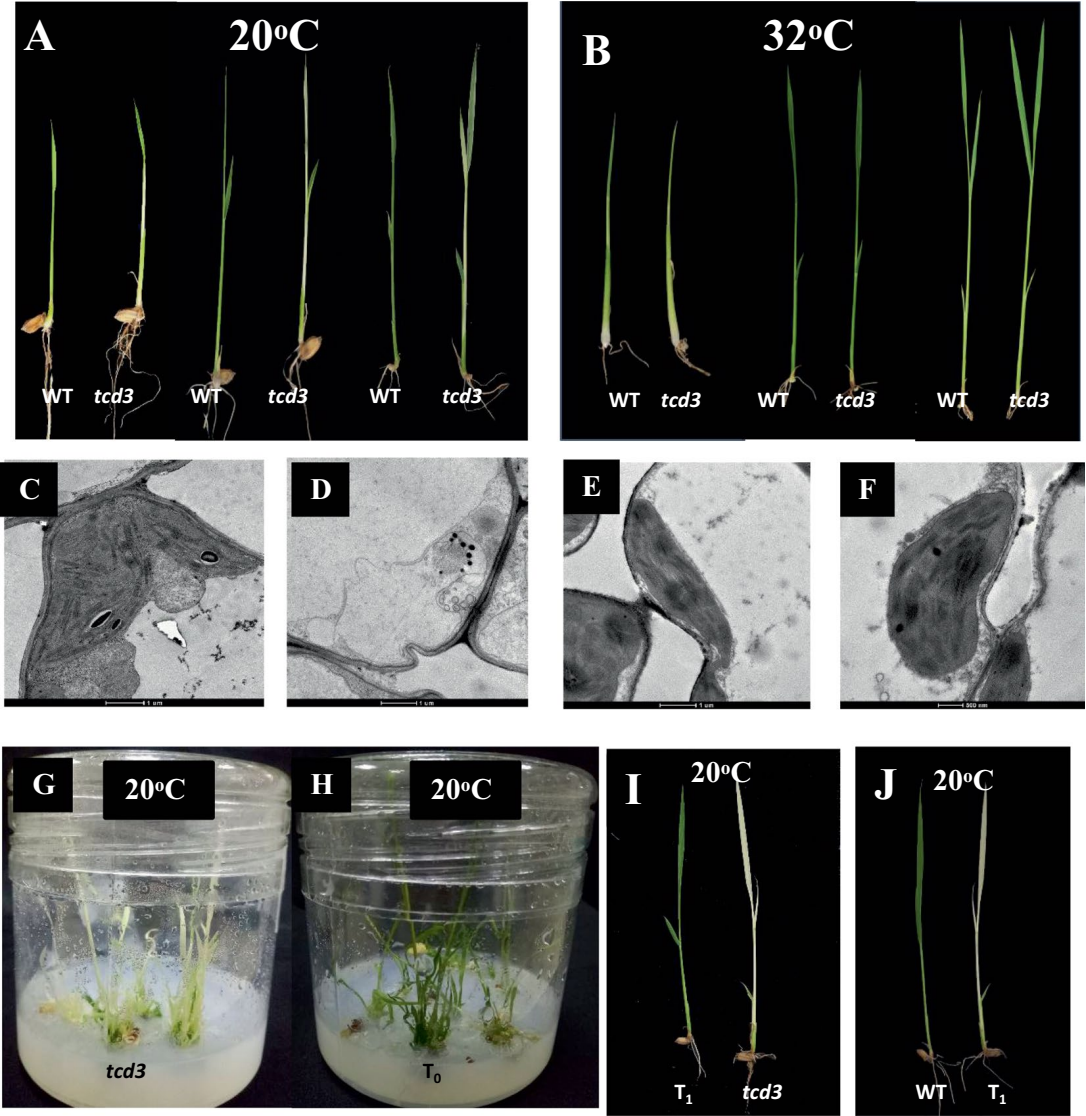
Characters of the *tcd3* mutant and WT plants; seedling leaf color in WT and *tcd3* at 20° (**A)** and 32° (**B**); chloroplast structure in WT at 20° (**C**) and 32° (**E**) and chloroplast structure in *tcd3* at 20° (**D**) and 32° (**F**); T_o_ complemented plants in the *tcd3* mutant (**G**) and control (**H**) backgrounds at 20° of the temperature of differentiation; segregation of T1 plants obtained from transgenic T0 plants transformed with pCAMBIA1301-TCD3 (**I**) and by CRISPR/Cas9 genome editing (**J**).

**Figure 2:**
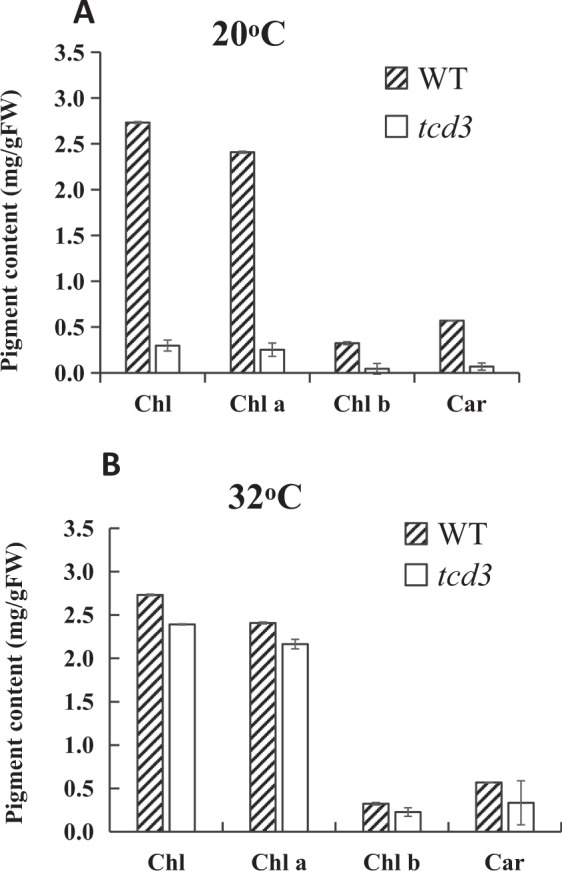
Pigment contents in the third leaves of WT and *tcd3* grown at 20° (**A**) and 32° (**B**), Chl, total chlorophyll; Chl a, chlorophyll a, Chl b, chlorophyll b; Car, carotenoid.

We observed the leaf color of wild-type (WT) and *tcd3* seedlings grown at two different temperatures, 20 °C and 32 °C (Fig. 1A,B). The *tcd3* seedlings displayed yellowish white leaves and died after the 5-leaf stage when grown at 20°. However, when grown at 32 °C, the seedlings remained green, as did WT seedlings at both temperatures. These results indicate that the variation in leaf color of the *tcd3* mutant is controlled by temperature and that this mutant has low-temperature-sensitive characteristics. Consistent with this conclusion, the photosynthetic pigments chlorophyll (Chl) a and b and carotenoid (Car) were almost undetectable in *tcd3* seedlings grown at 20 °C (Fig. 2A), whereas there was only a slight difference in pigment levels between WT and *tcd3* at 32 °C (Fig. 2B). These results indicate that *tcd3* exhibits low levels of photosynthetic pigment biosynthesis at 20 °C, but can quickly recover to almost WT levels at 32 °C. As expected, the structure of WT chloroplasts was complete in seedlings grown at both 32 °C and 20 °C. However, at 20 °C, almost no intact chloroplasts and numerous bubble-like structures were observed in *tcd3* (Fig. 1D), whereas *tcd3* at 32 °C and WT chloroplasts appeared similar (Fig. 1C,E,F). These results indicate that the *tcd3* mutation blocks chloroplast development under cold stress. Interestingly, under field conditions, the leaf Chl contents in *tcd3* plants were indistinguishable from those of WT plants (Fig. S1). In addition, except for a significant reduction in panicle number and grain number, no obvious differences in other traits were observed between *tcd3* and WT plants (Fig. S2), implying that the *tcd3* mutation has a somewhat negative affect on the later stages of plant growth.

### Map-based cloning of *TCD3*

**Figure 3:**
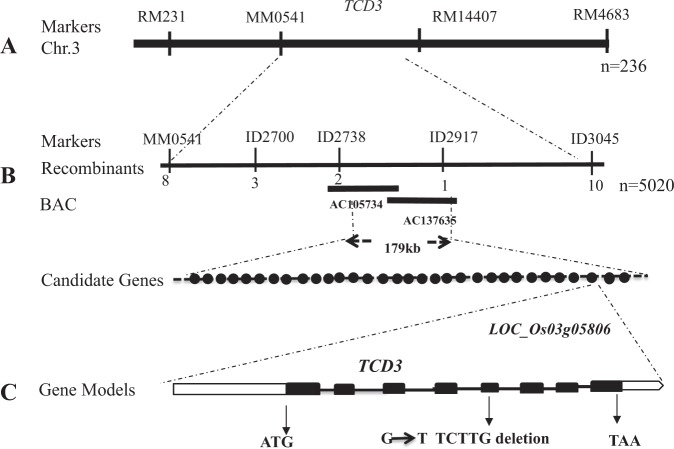
Cloning of the *TCD3* gene; (**A**), *TCD3* was initially localized to a region between SSR markers MM0541 and RM14407 on chromosome 3 using 236 F_2_ mutant individuals; (**B**), *TCD3* was narrowed down to a 179 kb region between ID2738 and ID2917 on BAC clones AC105734 and AC137635 using 5020 F2 mutant individuals; (**C**), gene model of *TCD3*.

Genetic analysis showed that the *tcd3* mutant trait was caused by a recessive mutation based on the approximately 3 (green): 1 (yellowish white) ratio for phenotypic segregation in an F_2_ population generated from Peiai64S/*tcd3* (Table S1). As a first step, we selected 236 mutant individuals in the F_2_ population and mapped *TCD3* between markers MM0541 and RM14407 on chromosome 3 (Fig. 3A). To further fine-map *TCD3*, we examined 5020 F_2_ mutant individuals and developed five InDel molecular markers (Table S2). Ultimately, *TCD3* was localized to a 179-kb region between markers ID2738 and ID2917 (Fig. 3B) spanning two BACs (AC105734, AC137635). Based on the Rice Genome Annotation Project (http://www.rice.plantbiology.msu.edu/), 32 candidate genes are predicted to be present in the target region, including one encoding a pseudouridine (Ψ) synthase (*LOC_Os03g05806*). We sequenced and analyzed all candidate genes and found only a G-to-A mutation at the 50^th^ base pair (bp) and a 5-bp deletion mutation (TCTTG) at bp 51 to 55 in the fifth exon of *LOC_Os03g05806* (Fig. 3C), which led to a premature translational stop and a frame-shift mutation, respectively.

### Complementation and knockout of *TCD3*

To verify that the yellowish white phenotype of *tcd3* at 20 °C is due to the mutation of *LOC_Os3g05806*, we performed genetic complementation of the *tcd3* mutant and CRISPR/Cas9 genome editing of WT plants. In the complementation test, to quickly obtain results, we induced the differentiation of rice callus at 20 °C. The resulting To transgenic seedlings harboring pCAMBIA1301:TCD3 appeared green (Fig. 1H), whereas non-transgenic seedlings remained yellow (Fig. 1G), indicating that *LOC_Os3g05806* can rescue the mutant phenotype. In addition, we obtained 19 independent transgenic (T_0_) plants transformed with pCAMBIA1301:TCD3. At 20 °C, we detected segregation of the mutant phenotype in the transgenic plant population (T_1_) and found that all green seedlings contained the transgene (Fig. 1I). As controls, we produced 14 independent lines transformed with the empty vector pCAMBIA1301, which failed to rescue the *tcd3* mutant phenotype. Via CRISPR/Cas9, we obtained 13 transgenic T_0_ plants carrying one editing mutation of the 1-bp (G) deletion at 89 bp from the ATG start codon in *TCD3* (Fig. S3). Importantly, all homozygous genome-edited T_1_ lines exhibited the same mutant phenotype as *tcd3* mutant at 20 °C (Fig. 1J). Taken together, these results confirm that *LOC_Os3g05806* is *TCD3*.

### Expression analysis and subcellular localization of TCD3

**Figure 4:**
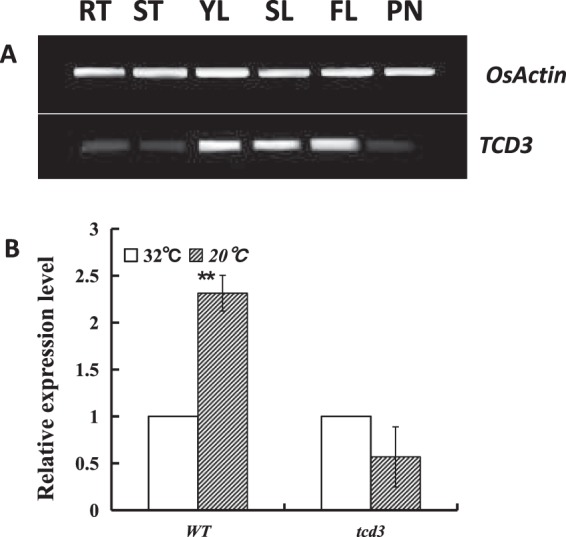
Transcript levels of *TCD3* in various tissues (**A**) and at different temperatures (**B**) determined by RT-PCR analysis; **YR**, young-seedling roots; **ST**, young-seedling stems; **YL**, young-seedling leaves; **SL**, the second leaf from the top; **FL**, flag leaf at heading; PN, young panicles. *OsActin* was used as a control (28 cycles for *OsActin*, 35 cycles for *TCD3*; Transcript levels (B) of *TCD3* in WT and *tcd3* at the 3-leaf stage at 20° and 32°, *OsActin* was used as a control for qPCR. Data are means ± SD (n = 3). Asterisks indicate statistically significant difference compared with WT. **P < 0.01 by Student’s t-test.

We examined the mRNA levels of *TCD3* by quantitative RT-PCR in various organs, including seedlings, roots, stems, leaves, and panicles, finding that *TCD3* was mainly expressed in leaves. Lower expression levels were also detected in roots, stems, and panicles (Fig. 4A). These results support the notion that TCD3 plays an important role in leaf development, which is consistent with the rice gene expression profiling data (http://ricexpro.dna.affrc.go.jp). To further investigate the effects of temperature on *TCD3* expression, we measured *TCD3* transcript levels in *tcd3* and WT seedlings at the 3-leaf stage grow, indicating that *TCD3* is a cold-inducible gene.

**Figure 5:**
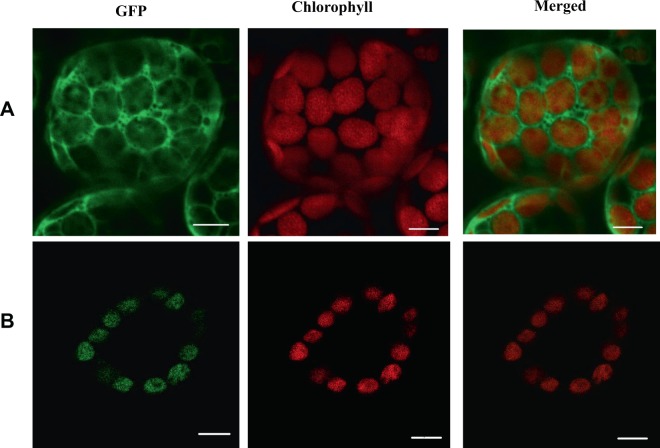
Subcellular localization of TCD3; (**A**) Empty GFP vector without a specific targeting sequence; (**B**) TCD3-GFP fusion protein; the scale bar represents 20 μm.

The *TCD3* protein was predicted to be localized to the chloroplast according to TargetP (http://www.cbs.dtu.dk/services/TargetP/)^16^. To determine the actual subcellular localization of TCD3, we transiently expressed a constitutively transcribed TCD3:GFP fusion construct (35 S:TCD3:GFP) in tobacco cells. The GFP signals from TCD3:GFP were localized to chloroplasts (Fig. 5B). These findings confirm that TCD3 is localized to the chloroplast and is induced under cold stress.

### Characterization of TCD3 protein by bioinformatic analysis

*TCD3* consists of eight exons and nine introns (Fig. 3C) and encodes a 415 amino-acid protein with a molecular mass of approximately 45 kDa. A search of the Pfam database^17^ revealed that TCD3 not only contains a chloroplast transit peptide (CTP; 70 aa), but it also contains an S4 domain and a Ψ synthase domain (Fig. S4A). The *tcd3* mutation site occurred in the Ψ synthase domain, which led to a change in the protein sequence and the destruction of its three-dimensional structure (Fig. S4B).

In addition, we searched various databases for sequences with similarity to each of the 11 *E. coli* Ψ synthases and found 22 homologous sequences in rice (Fig. S5). A phylogenetic tree showing the relationships of *E. coli* Ψ synthases, *Arabidopsis thaliana* SVR1 (At2g39140; involved in chloroplast rRNA processing)^15^, and Chlamydomonas Ψ synthase (Maa2)^14^ is shown in Fig. S6. Based on the results of phylogenetic analysis, TCD3 belongs to the RsuA family and shares the greatest sequence similarity with SVR1 (Fig. S5).

**Figure 6:**
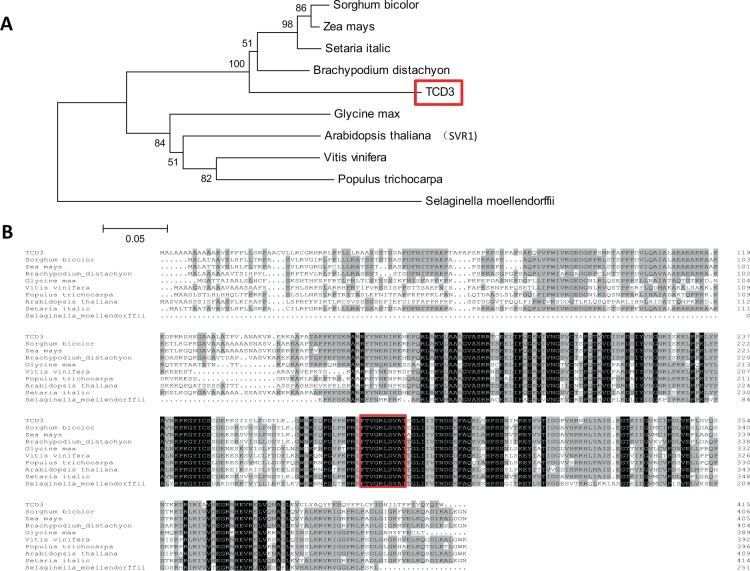
Phylogenic analysis of TCD3 and its homologs (**A**); Protein sequences are from *Sorghum bicolor (*8080543), *Zea mays* (100285657), *Setaria italic* (101780537, *Brachypodium distachyon (*100846238), *Arabidopsis thaliana (*At2g39140), *Glycine max* (100808046), *Vitis vinifera* (100247893), *Populus trichocarpa* (POPTR_0008s03540g), and *Selaginella moellendorffii* (SELMODRAFT_45172). Scale represents percentage substitutions per site. The rooted tree is based on multiple sequence alignment using MAFFT and was generated with MEGA 6; Amino acid sequence alignment of TCD3 with its nine homologs (**B**). Fully and partially conserved amino acids are shaded in black and gray, respectively. The homologous comparison is based on multiple sequence alignment using MAFFT and was generated with the program DNAMAN8.

Further study showed that TCD3 is conserved in higher plants, especially in millet, maize, two ear small grass, and sorghum, with similarities of 79.3%, 76.5%, 77.5%, and 79.3%, respectively (Fig. 6B). Phylogenetic analyses showed that the TCD3 homologs could be clearly divided into two groups: monocotyledons and dicotyledons, which conforms with the taxonomy (Fig. 6A).

### The disruption of *TCD3* alters the transcript levels of related genes

**Figure 7:**
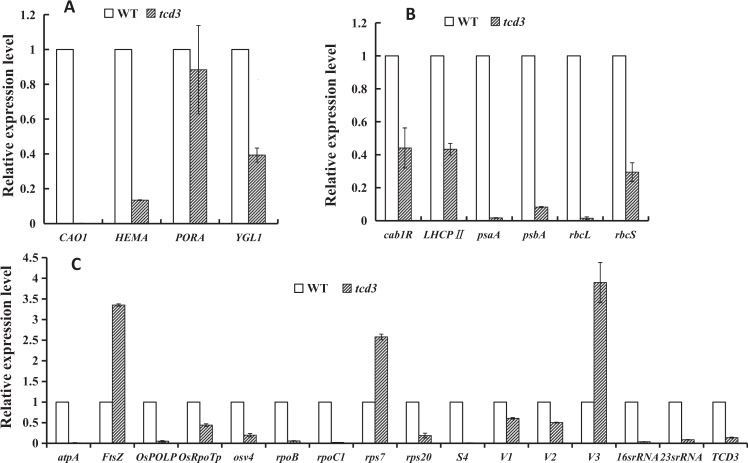
qRT-PCR analysis of genes associated with Chl biosynthesis **(A**), photosynthesis **(B**), and chloroplast development in wild type (WT) and *tcd3* at the 3-leaf stage at 20°; the relative expression level of each gene in WT and *tcd3* was analyzed by qRT-PCR and normalized using *OsActin* as an internal control. Data are means ± SD (n = 3).

**Figure 8:**
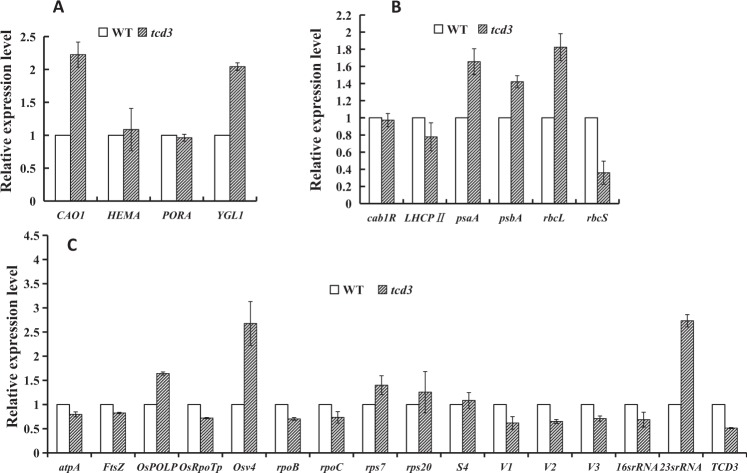
qRT-PCR analysis of genes associated with Chl biosynthesis **(A**), photosynthesis **(B**), and chloroplast development in wild type (WT) and *tcd3* at the 3-leaf stage at 32°; the relative expression level of each gene in WT and *tcd3* was analyzed by qRT-PCR and normalized using *OsActin* as an internal control. Data are means ± SD (n = 3).

To determine the effect of the *tcd3* mutation on the expression of genes related to chloroplast development and to explore the underlying pathway, we carried out RT-qPCR analysis of genes involved in Chl biosynthesis, photosynthesis, and chloroplast development in rice. At both high and low temperatures, low levels of *TCD3* transcripts were detected in *tcd3*, confirming that the *tcd3* mutation blocks the transcription of this gene (Figs. 7C and 8C). At 20 °C, Chl biosynthesis (*CAO*, *YGL1*, *Cab1R*) and photosynthesis (*rbcS, rbcL*, *psa*A, *psbA*, *LHCPII*)-related genes were significantly downregulated in *tcd3*, although *PORA* (Fig. 7A–C) was not, which is consistent with the chlorosis symptoms observed at low temperatures. Among chloroplast development-associated genes, except for *Ftsz* (encoding a component of the plastid division machinery)^18,19^, *V3* (*RNRL*, encoding the large subunit of ribonucleotide reductase)^18^, and *rps7* (encoding the small subunit ribosomal protein S7), which were upregulated in *tcd3* at 20 °C, all remaining genes were strongly downregulated in the mutant. In particular, several genes were severely downregulated, including *atpA*, encoding an ATP synthase subunit^20^; *OsPOLP*, encoding a plastidial DNA polymerase^22^; *rpoC1*, encoding RNA polymerase β‘ subunit; S4, encoding ribosomal protein subunit S4; and *16SrRNA* and *23SrRNA*, encoding the large (16S) and small (23S) subunits of chloroplast ribosomes, respectively. Notably, however, the transcript levels of all up- and downregulated genes in *tcd3* at 20 °C were restored to WT levels at 32 °C, and other genes were also expressed at WT levels when grown at 32 °C (Fig. 8A–C) (within a twofold range), which corresponds with the thermo-sensitivity of the mutant phenotype. In summary, the *tcd3* mutation reduces the mRNA levels of certain genes involved in Chl biosynthesis and photosynthesis, as well as chloroplast development, at low temperatures.

## Discussion

In the current study, we identified and characterized TCD3, a pseudouridine (Ψ) synthase required for chloroplast development at low temperatures in rice. Plants with a loss of function of *TCD3* produced imperfect chloroplasts and exhibited a Chl-deficient phenotype at low temperatures, resulting from the abnormal expression of genes associated with Chl biosynthesis, photosynthesis, and chloroplast development. Our results provide evidence that cold-induced *TCD3* plays an important role in chloroplast development in rice at low temperatures.

### TCD3 regulates the first stage of chloroplast development in rice at low temperatures

Chloroplast development in rice, which is coordinately regulated by plastid and nuclear genes, can be divided into three stages^2–4^. The first stage involves *OsPOLP1* (encoding a plastidial DNA polymerase) and *FtsZ* (encoding a component of the plastid division machinery)^18,20^. The second stage involves *OsRpoTp* (encoding a NEP), *V2* (encoding a plastidial guanylate kinase), and *rpoA* (encoding a PEP subunit)^19–22^. The third stage involves PEP-transcribed plastid genes (e.g., *psbA, rbcL*)^3^. Other mutants with phenotypes similar to the cold-sensitive phenotype of *tcd3* have also been reported, including the *v1*, *v2*, *v3*, *tcd9, osv4*, *tcd5*, *tcd10*, and *tcd11, tsv3 and tcm1* mutants^19,23–30^. In detail, *V1* encodes the chloroplast-localized protein NUS1, which is involved in regulating chloroplast RNA metabolism at the second stage of chloroplast development^3^. *V2*, encoding plastid or mitochondrial guanylate kinase (pt/GK), is required for the second stage of chloroplast development^19^. *V3*, encoding the large subunit of ribonucleotide reductase, is required for the first stage of chloroplast development^31^. More recently, we identified five additional genes that are essential for chloroplast development at low temperature: *TCD9*, encoding the α subunit of chaperonin protein 60^25^; *OsV4* and *TCD10*, both encoding PPR proteins^26,28^; *TCD5*, encoding a monooxygenase^32^; *TCD11*, encoding plastid ribosomal protein S6^27^; and *TSV3*, encoding Spo0B-associated GTP-binding (Obg) protein^29^. Among these, *OsV4*, *TCD5*, *TCD10*, and *TCD11* participate in regulating the second stage of chloroplast development, while *TCD9* and *TSV3* participate in the first stage. In this study, the high levels of *TCD3* transcript (Fig. 4B) detected at 20 °C indicate that *TCD3* is a cold-inducible gene that is required for chloroplast development under cold stress. Notably, in *tcd3* plants at 20 °C, we detected severely reduced transcript levels (Fig. 8C) of *OsPOLP1*, which is involved in the first step of chloroplast development^20,22^, indicating that TCD3 regulates the first stage of chloroplast development in rice. Similarly, the transcript levels of *OsRpoTp* (encoding a NEP), as well as NEP-dependent genes (*16S rRNA, 23S rRNA, rps20, rpoB, rpoC1, V1, V2*), PEP- and NEP-dependent *atpA* (which functions in the second stage of chloroplast development), and PEP-regulated plastid genes (*Cab1R, LHCPII, psaA, psbA, rbcL*, and *rbcS*, which function in the third stage of chloroplast development) were also severely reduced in the mutant under cold stress (Fig. 7). Perhaps the increased mRNA levels of *Ftsz* and *V3* during the first stage of chloroplast development and of *rps7* during the second stage are due to feedback effects. In addition, the recovery of the expression levels of all affected genes at 20 °C to WT levels in *tcd3* at 32 °C likely accounts for the normal phenotype of the mutant at 32 °C (Fig. 8A–C). The low expression level of *TCD3* at 32 °C and the high expression level at 20 °C suggest that TCD3 is indispensable for chloroplast development at low temperatures but not at high temperatures. Therefore, we conclude that TCD3 functions in the first stage of chloroplast development at low temperatures.

### Possible role of TCD3 in chloroplast rRNA and tRNA metabolism at low temperatures

In this study, we identified the first Ψ synthase, TCD3, in rice. As mentioned above, Ψ synthases can be divided into five families. Based on the presence of an S4 domain and a Ψ synthase domain (Fig. S5A) and the results of homology analysis, TCD3 belongs to the RsuA family, members of which isomerize U (uridine) 516 in rRNA^11^. *TCD3* proteins also share a conserved nine-amino acid motif (GRLDVATSG; Fig. 6B) at the active site that includes a universally conserved Asp (D) residue^33^. In addition, the most similar protein to TCD3 is *Arabidopsis thaliana* SVR1 (60.6% sequence similarity; Fig. S6), which shares the same domains. In addition to playing a role in uridine isomerization, SVR1 is also required for proper chloroplast rRNA processing and tRNA metabolism^15^. Surprisingly, like 16 S rRNA and 23 S rRNA (both related to chloroplast rRNA processing), the levels of nuclear (*rbcS*, *LhcpII*) and plastid (*rbcL, psaA, atpA*) gene expression were severely impaired in both *svr1*^15^ and *tcd3* (Fig. 7C). In *tcd3* plants, the transcript levels of two other ribosomal protein genes (*rps20* and *S4*) were also severely reduced. Thus, TCD3, like SVR1, might play a role in chloroplast rRNA processing and tRNA metabolism at low temperatures. Therefore, under cold stress, the *tcd3* mutation affects chloroplast rRNA processing and tRNA metabolism, which in turn leads to the production of yellowish white leaves Accordingly, the loss of TCD3-mediated *OsPOLP1*-*16S*-*23S rRNA* mRNA expression might lead to the thermo-sensitive phenotype observed under cold stress. Why does *TCD3* exhibit thermo-sensitivity? Perhaps different Ψ synthases utilize different mechanisms during environmental responses. For example, *Chlamydomonas reinhardtii Maa2*, which is homologous to *LOC_Os3g26440* (Fig. S5), is highly photosensitive^14^, in contrast to the thermo-sensitivity of *TCD3*. The findings highlight the notion that even highly conserved genes within the same species or across species might play more diverse, complex roles than previously recognized. Under high temperature conditions, perhaps *TCD3* is not required for chloroplast development because the function of *TCD3* is replaced by that of other homologous genes (Fig. S5). Finally, our observations provide evidence for the versatile roles of plant Ψ synthases in development. Taken together, our results indicate that *TCD3* is required for chloroplast development during the early stages of leaf development in rice under cold stress.

## Materials and methods

### Plant materials and growth conditions

The thermo-sensitive chlorophyll-deficient *tcd3* mutant was derived from *japonica* rice variety Jiahua 1 (WT) treated with ^60^Co gamma-radiation. This thermo-sensitive leaf color mutant exhibits leaf yellowing at low temperatures. The F_2_ mapping population was generated from a cross between Pei’ai 64S (*indica*) and *tcd3*. All rice seedlings were grown in growth chambers under controlled conditions with a 12-h-dark/12-h-light cycle at 20 °C or 32 °C.

### Phenotypic characterization and measurement of photosynthetic pigments

Germinated seeds of *tcd3* and Jiahua 1 (WT) were sown on soils under controlled conditions as described above at 20 °C or 32 °C. The 3^rd^ leaves (200 mg) from plants at the three-leaf stage were homogenized in 10 mL of 100% acetone. The absorbance value of the supernatant at 470, 645, and 663 nm was determined by spectrophotometry (Beckman Coulter, Danvers, MA, USA). The chlorophyll (Chl) and carotenoid (Car) contents were determined by spectrophotometry as described by Arnon^34^ and Wellburn^35^. In addition, WT and *tcd3* plants were grown in a paddy field at Shanghai Normal University, China, in 2010. Leaf Chl SPAD values (Fig. S1) were measured using a chlorophyll meter (SPAD-502, Minolta Co. Ltd., Japan), a nondestructive, rapid method for estimating photosynthetic pigment levels^36–38^. The SPAD values were measured every week from transplantation (summer) to the heading (autumn) stage. Various yield-associated traits (Fig. S2) were investigated at maturity.

### Observation of chloroplast structure by transmission electron microscopy (TEM)

Chloroplast development was observed in the third leaves of WT and *tcd3* plants using TEM as described elsewhere^39^ with minor modifications. Briefly, the third leaves were sampled from seedlings at the 3-leaf-stage grown at 20 °C and 32 °C. Transverse leaf sections were fixed in 2.5% glutaraldehyde solution at 4° for 4 h, rinsed and incubated overnight in 1% w/v OsO_4_ at 4°, and embedded in Epon 812 resin. The samples were stained again and examined under a Hitachi-7650 transmission electron microscope.

### Positional cloning of *TCD3*

Genomic DNA was extracted from rice leaves using an improved CTAB method^40^. Initially, 236 F_2_ recessive seedlings from the cross Pei’ai 64 S/*tcd3* were used to identify the molecular markers linked to the mutant gene. Subsequently, 5020 F_2_ plants showing the mutant phenotype were used for fine mapping. New molecular markers were designed by comparing the sequences of 93–11^41^ and Nipponbare^42^ from NCBI (http://www.ncbi.nlm.nih.gov); the markers are listed in Table S2.

### Complementation of the *tcd3* mutant

For the complementation test, genomic DNA was extracted from WT plants and used as a PCR template with the primer pair TCD3F: 5′-GGGGTACCGCCCATACAGATCCTCG-3′ (*Kpn*I) and TCD3R: 5′-GCGTCGACTTTCAGCAAACCCCATG-3′ (*Sal*I), amplifying a fragment (6.9 kb) containing the target gene (*TCD3*) and the 1.5 kb upstream and 0.35 kb downstream sequences. The PCR products were cloned into the pMD18-T vector (TaKaRa, Dalian, China). The pMD18T-*TCD3* construct was digested with *Kpn*I and *Sal*I and ligated into the *Kpn*I and *Sal*I site of binary vector pCAMBIA1301 (CAMBIA, http://www.cambia.org.au). The resulting pCAMBIA1301:*TCD3* vector was transferred into *Agrobacterium tumefaciens* strain EHA105 and introduced into the *tcd3* mutant by Agrobacterium-mediated transformation according to previously published methods^43^, except that the temperature used for in *vitro* plant differentiation was 20 °C. The genotypes of the transgenic seedlings were determined by PCR amplification of the hygromycin phosphotransferase gene (*hpt*) with primers HPTF (5′-GGAGCATATACGCCCGGAGT-3′) and HPTR (5′-GTTTATCGGCACTTTGCATCG-3′) and primers GUSF (5′-GGGATCCATCGCAGCGTAATG-3′) and GUSR (5′-GCCGACAGCAGCAGTTTCATC-3′) as selection markers. The resulting T_0_ transgenic seedlings were grown in a paddy field after screening for hygromycin-tolerant plants and confirmation by DNA sequencing. All T_1_ seedlings were grown at 20° and used to examine the segregation of the mutant phenotype.

### Knockout of *TCD3*

To experimentally affirm that *TCD3* is responsible for the phenotypic changes observed in *tcd3*, CRISPR/Cas9 genome editing was performed on WT plants. To generate the Cas9 targeting construct for *TCD3* using CRISPR Primer Designer software(http://www.crispr.dbcls.jp/)^44^, annealed gRNA oligonucleotide pairs with recognition sequences were designed (F1: 5′-GCCGGACTTCAAC ATCACCTTCG-3′, R1: 5′-AAACCGAAGGTGATGTTGAAGTC-3′; F2: GCCGGACTTCAACATCACCTTCG, R2: AAACCGAAGGTGATGTTGAAGTC; F3: GCCGCCTGCGTGCTCCTCCGCTG, R3: AAACCAGCGG AGGAGCACGCAGG; F4: GCCGGCGGAGGCGGAGGCCAAGA). The recognition sequences were inserted into the region between the OsU6 promoter and the gRNA scaffolds (from the pYLgRNA-OsU6 vector) of the Cas9 expression backbone vector (pYL-CRISPR/Cas9-MH) at the *Bsa*I sites as previously described^45^. The resulting plasmid (CRISPR/Cas9 expression) and the empty vector were introduced into *Agrobacterium* strain EHA105 and used to infect calli from WT plants^43^. The resulting T_1_ seedlings were grown and used to examine the segregation of the mutant phenotype at 20 °C.

### Subcellular localization

To investigate the subcellular localization of TCD3, cDNA fragments encoding the 144 amino-acid N-terminal region of *TCD3* were amplified from total RNA extracts using following primers (5′-GAAGATCTATGGCCCTCGCCGCCGCCGCCGCCGCCGCG-3′ and 5′-GGGGTACCCCCCTCTCCCTCACCTTGGCAT-3′) and introduced into vector pMON530-GFP at the *Bgl*II and *Kpn*I sites (the underlined sequences represent cleavage sites). The resulting pMON530:CaMV35S:TCD3-GFP plasmid was verified by sequencing and introduced into *Agrobacterium* strain EHA105. The localization of TCD3 was investigated by transient expression analysis of the GFP fusion protein in tobacco (*Nicotiana tabacum*) cells via confocal microscopy as described previously^25^. GFP fluorescence in the transformed protoplasts was imaged by confocal laser-scanning microscopy (LSM5 PASCAL; ZEISS, http://www.zeiss.com).

### RT-PCR and quantitative RT-PCR (qRT-PCR) analysis

Total RNA from WT plants was extracted from seedling roots (ST), young stems (YS), the third leaves of plants at the 3-leaf stage, the second leaves (SL) from the tops of plants, and flag leaves (FL) and young panicles (PN) from plants at the heading stage using an RNA Prep Pure Plant kit (Tiangen Co., Beijing, China) to investigate the tissue-specific expression patterns of *TCD3*. The RNA was reversely transcribed into cDNA and used for RT-PCR as described previously^25^. In addition, RNA from the third leaves of WT and *tcd3* seedlings at the 3-leaf stage grown at 20 °C and 32 °C was extracted as described above and used to measure the transcript levels of *TCD3* as well as Chl biosynthesis, chloroplast development, and photosynthesis-associated genes (*atpA, Cab1R, CAO, FtsZ, HEMA, LHCP*II*, OsPOLP, OsRpoTp, OsV4, PORA, psaA, psbA, rbcL, rbcS, rpoB, rpoC, rps7, rps20, RNRL, S4, V1, V2, YGL-1, 16SrRNA, 23SrRNA*) in rice. The qPCR was performed using a SYBR Premix Ex Taq^TM^ kit (TaKaRa) on an ABI7500 Real-Time PCR System (Applied Biosystems; http://www.appliedbiosystems.com), and the relative quantification of gene expression data was performed as described in Livak and Schmittgen^46^. The specific primers for qPCR were partially referred to Wu *et al*.^47^ based on sequences from NCBI; the primers are listed in Table S3. The rice *OsActin* gene was used as a reference gene.

### Sequence and phylogenetic analyses

Gene prediction and structure analysis were carried out using the GRAMENE database (http://www.gramene.org). Homologs of TCD3 were identified by BLASTP analysis against the National Center for Biotechnology Information database (http://www.ncbi.nlm.nih.gov) and subjected to multiple sequence alignment using DNAMAN version 6.0 (Lynnon Biosoft, USA). The signal peptide was predicted with SignalP version 2.0^47^. The phylogenetic tree was constructed using MEGA version 6 software (http://www.megasoftware.net).

## Supplementary information

Supplementary information.
