# Supplementary material for: Chloroplast development at low temperature requires the pseudouridine synthase gene TCD3 in rice
Source: Sci Rep. 2020 May 22;10:8518. doi: 10.1038/s41598-020-65467-2 (PMC7244722; doi:10.1038/s41598-020-65467-2)
Supplement: Supplementary file 1 — Supplementary information. [file 41598_2020_65467_MOESM1_ESM.pdf]

Chloroplast development at low temperature requires the pseudouridine synthase gene *TCD3* in rice

Dongzhi Lin· Rongrong Kong· Lu Chen· Yulu Wang· Lanlan Wu· Jianlong Xu·Zhongze Piao· Gangseob Lee· Yanjun Dong

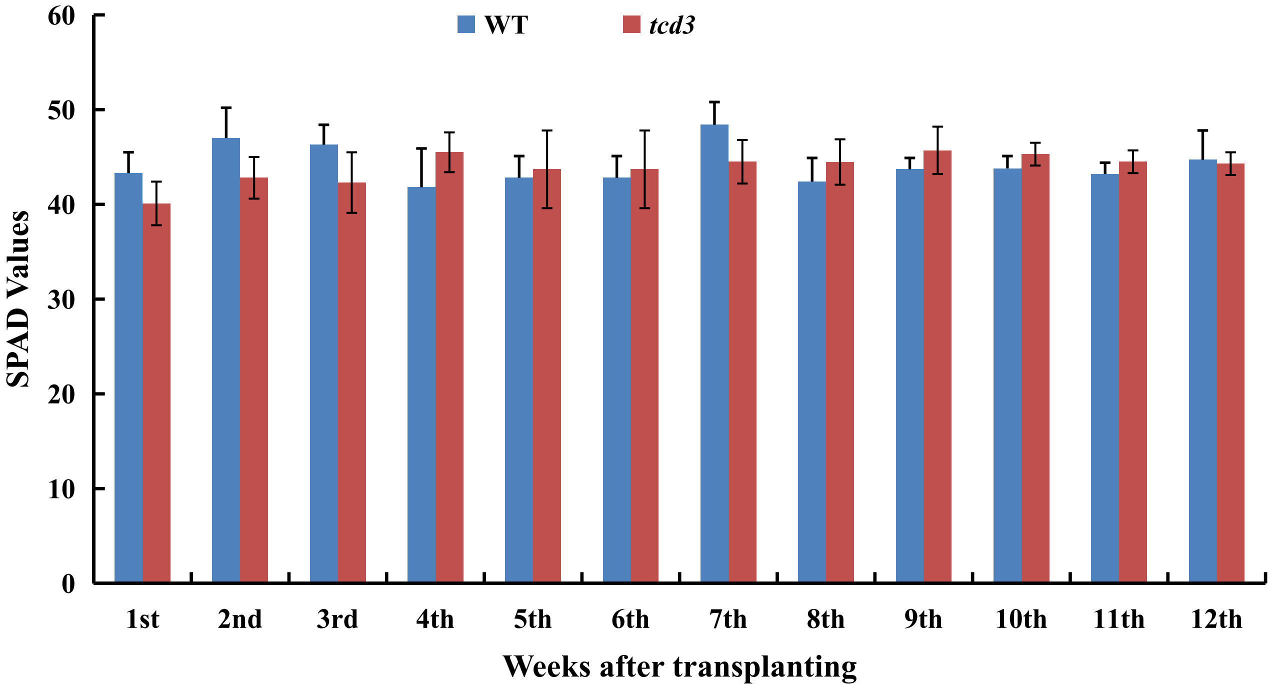

Fig. S1 Changes in leaf chlorophyll SPAD values from transplantation to maturity

Chloroplast development at low temperature requires the pseudouridine synthase gene *TCD3* in rice

Dongzhi Lin· Rongrong Kong· Lu Chen· Yulu Wang· Lanlan Wu· Jianlong Xu·Zhongze Piao· Gangseob Lee· Yanjun Dong

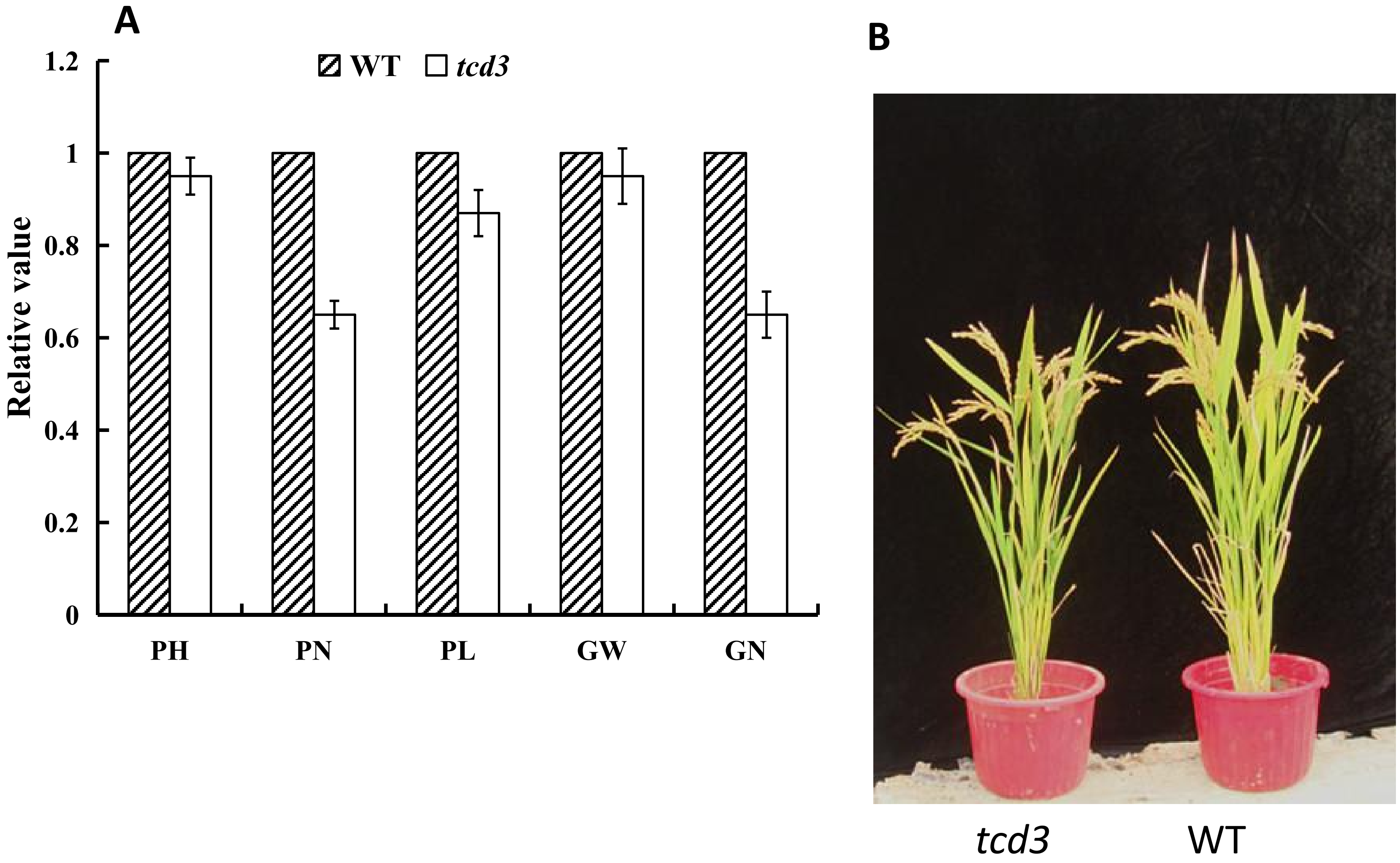

Fig. S2 Comparison of panicle-related traits between *tcd3* and wild type (grown in Shanghai, China); PH, Panicle high PN, Panicle number; PL, Panicle length (cm); GW, 1000-grain weight (g); GN, grain number.

Chloroplast development at low temperature requires the pseudouridine synthase gene *TCD3* in rice

Dongzhi Lin· Rongrong Kong· Lu Chen· Yulu Wang· Lanlan Wu· Jianlong Xu·Zhongze Piao· Gangseob Lee· Yanjun Dong

```
WT        ATGGCCCTCGCCGCCGCCGCCGCCGCCGCGGCAGCGGCCGTCTCCTTCCCGCCCTTGCTCTCCCGTCCCGCCGCCTGCGTGCTCCTCCGCTGCGGCCGCCACCGCCGCCTCCCTCCCCTC   120
TCD3-     ATGGCCCTCGCCGCCGCCGCCGCCGCCGCGGCAGCGGCCGTCTCCTTCCCGCCCTTGCTCTCCCGTCCCGCCGCCTGCGTGCTCCTCC.CTGCGGCCGCCACCGCCGCCTCCCTCCCCTC   119
Consensuatggccctcgccgccgccgccgccgccgcggcagcggccgtctccttcccgcccttgctctcccgtcccgccgcctgcgtgctcctcc ctgcggccgccaccgccgcctccctcccctc

WT        CTCCTCCGCGCCGCCTCCTCCTCCACCTCCAGCGCCCCGGACTTCAACATCACCTTCGCGGAGCCCACCGCCCCCGCCCCCTCGAAACCCAAACCCTCATCCCCCGCCCCGTCCGCGGAG   240
TCD3-     CTCCTCCGCGCCGCCTCCTCCTCCACCTCCAGCGCCCCGGACTTCAACATCACCTTCGCGGAGCCCACCGCCCCCGCCCCCTCGAAACCCAAACCCTCATCCCCCGCCCCGTCCGCGGAG   239
Consensuctcctccgcgccgcctcctcctccacctccagcgccccggacttcaacatcaccttcgcggagcccaccgcccccgccccctcgaaacccaaaccctcatcccccgccccgtccgcggag

WT        CAGGTGGTGCCGTGGATCGTGCGTGGGGAGGACGGCAAGCCGTCCATGCGCACCGCGCCGCCGCCGGACGTCCTCCAGGCCATTGCGCTGGCGGAGGCGGAGGCCAAGAAGGCCAAGAAG   360
TCD3-     CAGGTGGTGCCGTGGATCGTGCGTGGGGAGGACGGCAAGCCGTCCATGCGCACCGCGCCGCCGCCGGACGTCCTCCAGGCCATTGCGCTGGCGGAGGCGGAGGCCAAGAAGGCCAAGAAG   359
Consensucaggtggtgccgtggatcgtgcgtggggaggacggcaagccgtccatgcgcaccgcgccgccgccggacgtcctccaggccattgcgctggcggaggcggaggccaagaaggccaagaag

WT        GACCCCCGCAGGAGCCACAAGGGCGCCGCCGCCCTTGCCACCCCCGTCGCCAATGCCAAGGTGAGGGAGAGGAAGGCCGCCCCCGCGACGGCGCCGCCCAAGTTCTCCAAGGCCGCGAGG   480
TCD3-     GACCCCCGCAGGAGCCACAAGGGCGCCGCCGCCCTTGCCACCCCCGTCGCCAATGCCAAGGTGAGGGAGAGGAAGGCCGCCCCCGCGACGGCGCCGCCCAAGTTCTCCAAGGCCGCGAGG   479
Consensugacccccgcaggagccacaagggcgccgccgcccttgccacccccgtcgccaatgccaaggtgagggagaggaaggccgcccccgcgacggcgccgcccaagttctccaaggccgcgagg

WT        AGATTCTACAATGAGAATATCAAGGAGCACGAACCGCAGCGGCTCGCCAAGGTCCTTGCCGCGGCAGGAGTGGCCTCACGAAGGACATGCGAGGAGCTCATCTTCCAAGGGAAGGTGACC   600
TCD3-     AGATTCTACAATGAGAATATCAAGGAGCACGAACCGCAGCGGCTCGCCAAGGTCCTTGCCGCGGCAGGAGTGGCCTCACGAAGGACATGCGAGGAGCTCATCTTCCAAGGGAAGGTGACC   599
Consensuagattctacaatgagaatatcaaggagcacgaaccgcagcggctcgccaaggtccttgccgcggcaggagtggcctcacgaaggacatgcgaggagctcatcttccaagggaaggtgacc

WT        GTCAATGGTTCTGTCTGCACTGCACCCCAGACCAAAGTTGACATCTCAAAGGATTCTATCTATGTTAATGGAAACCGCATTTCCAAGAAGCTTCCTCCGAAGTTATATTTTGCTGTTAAC   720
TCD3-     GTCAATGGTTCTGTCTGCACTGCACCCCAGACCAAAGTTGACATCTCAAAGGATTCTATCTATGTTAATGGAAACCGCATTTCCAAGAAGCTTCCTCCGAAGTTATATTTTGCTGTTAAC   719
Consensugtcaatggttctgtctgcactgcaccccagaccaaagttgacatctcaaaggattctatctatgttaatggaaaccgcatttccaagaagcttcctccgaagttatattttgctgttaac

WT        AAGCCAAAAGGGTATATTTGTTCCTGTGGTGAACCAAAGTCCATTATCTCCTTATTTGATGACTATTTGAAGGGTTGGAACAAGATTCAACCAGGAGTACCAAAACCGCGCTTGTTTACT   840
TCD3-     AAGCCAAAAGGGTATATTTGTTCCTGTGGTGAACCAAAGTCCATTATCTCCTTATTTGATGACTATTTGAAGGGTTGGAACAAGATTCAACCAGGAGTACCAAAACCGCGCTTGTTTACT   839
Consensuaagccaaaagggtatatttgttcctgtggtgaaccaaagtccattatctccttatttgatgactatttgaagggttggaacaagattcaaccaggagtaccaaaaccgcgcttgtttact

WT        GTGGGTCGTCTTGATGTTGCTACAAGTGGTTTGATAATAATTACAAATGACGGTGAGTTTGCCCAGAAACTCTCACACCCTTCGTCAAATATAATGAAGGAATATGTGGTAACTATAGAT   960
TCD3-     GTGGGTCGTCTTGATGTTGCTACAAGTGGTTTGATAATAATTACAAATGACGGTGAGTTTGCCCAGAAACTCTCACACCCTTCGTCAAATATAATGAAGGAATATGTGGTAACTATAGAT   959
Consensugtgggtcgtcttgatgttgctacaagtggtttgataataattacaaatgacggtgagtttgcccagaaactctcacacccttcgtcaaatataatgaaggaatatgtggtaactatagat

WT        GGACCAGTGCACAGAAATCACCTTATAGCTATCAGTGAAGGCACAAAAATTGATGGCGTCAAATGTATACCTGATCTAGTGGAACCATTGGATGTACAATCCAATACAAAAAGGACCCGG  1080
TCD3-     GGACCAGTGCACAGAAATCACCTTATAGCTATCAGTGAAGGCACAAAAATTGATGGCGTCAAATGTATACCTGATCTAGTGGAACCATTGGATGTACAATCCAATACAAAAAGGACCCGG  1079
Consensuggaccagtgcacagaaatcaccttatagctatcagtgaaggcacaaaaattgatggcgtcaaatgtatacctgatctagtggaaccattggatgtacaatccaatacaaaaaggacccgg

WT        ATTAGAATAGCGGTTCATGAAGGAAGAAATCATGAAGTTCGTGAACTCGTGCAGAATGCAGGCCTAGAGGTTTGTTTGTATGCTCAATATTTTTTTTCTCAAGTTTTCCCTTTATGTTTT  1200
TCD3-     ATTAGAATAGCGGTTCATGAAGGAAGAAATCATGAAGTTCGTGAACTCGTGCAGAATGCAGGCCTAGAGGTTTGTTTGTATGCTCAATATTTTTTTTCTCAAGTTTTCCCTTTATGTTTT  1199
Consensuattagaatagcggttcatgaaggaagaaatcatgaagttcgtgaactcgtgcagaatgcaggcctagaggtttgtttgtatgctcaatattttttttctcaagttttccctttatgtttt

WT        ATTGATCATATCTTAACATTTCCCATTGTGCAATATCAAATTTGGTA                                                                          1247
TCD3-     ATTGATCATATCTTAACATTTCCCATTGTGCAATATCAAATTTGGTA                                                                          1246
Consensuattgatcatatcttaacatttcccattgtgcaatatcaaatttggta
```

Fig. S3 Full-length cDNA sequence of *TCD3* and mutation sites; the red box represents the knockout sequence in the CRISPR/Cas9 experiments.

Chloroplast development at low temperature requires the pseudouridine synthase gene *TCD3* in rice

Dongzhi Lin· Rongrong Kong· Lu Chen· Yulu Wang· Lanlan Wu· Jianlong Xu·Zhongze Piao· Gangseob Lee· Yanjun Dong

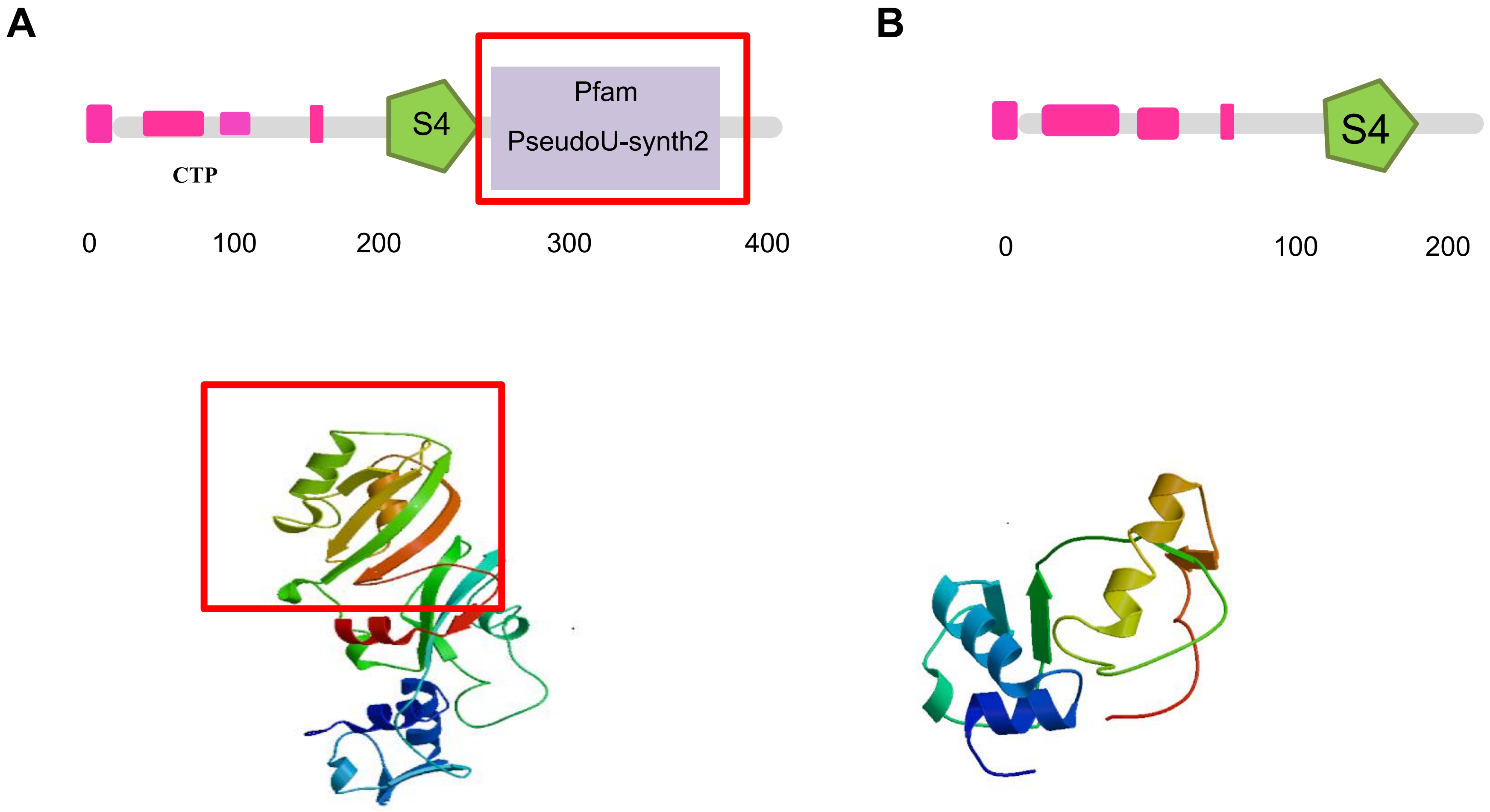

Fig. S4 3D structure of TCD3 predicted by SWISS-MODEL (http://swissmodel.expasy. org/). (A) TCD3 structure in wild type (WT). (B) TCD3 structure in *tcd3*.

Chloroplast development at low temperature requires the pseudouridine synthase gene *TCD3* in rice

Dongzhi Lin· Rongrong Kong· Lu Chen· Yulu Wang· Lanlan Wu· Jianlong Xu·Zhongze Piao· Gangseob Lee· Yanjun Dong

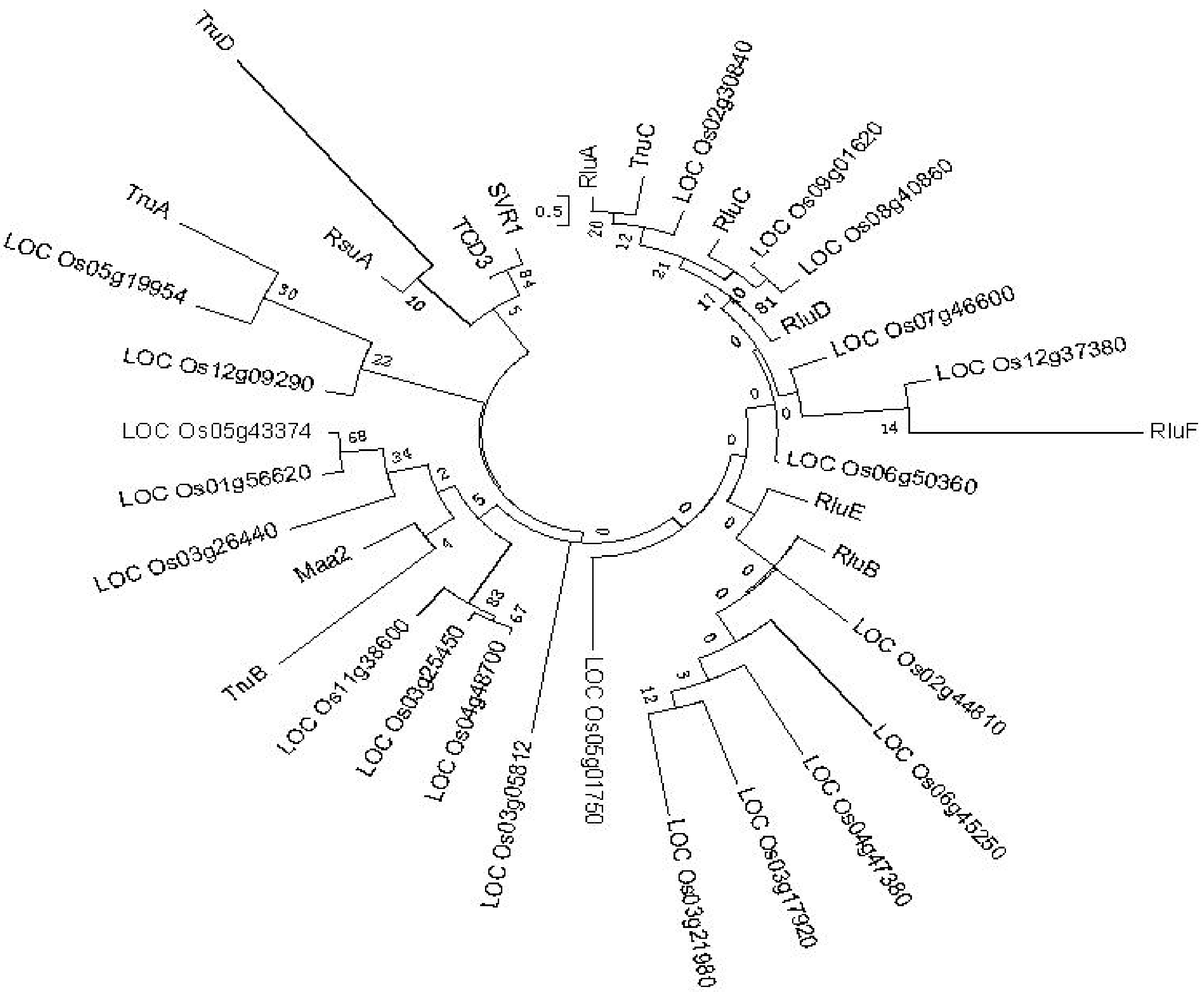

Fig. S5 Phylogenetic analysis of pseudouridine synthases. Phylogenetic tree of pseudouridine synthases from *E. coli* (Tru, Rlu, and Rsu). *Oryza*, *Arabidopsis* (SVR1), and Chlamydomonas (Maa2). Full-length protein sequences were obtained from the National Center for Biotechnology Information and The Rice Genome Annotation Project, aligned, and analyzed using MEGA 6.1.

Chloroplast development at low temperature requires the pseudouridine synthase gene *TCD3* in rice

Dongzhi Lin· Rongrong Kong· Lu Chen· Yulu Wang· Lanlan Wu· Jianlong Xu·Zhongze Piao· Gangseob Lee· Yanjun Dong

Table S1 Genetic segregation analysis of *tcd3* mutants in the F_2 population

Cross	Observed number of F_2 plants			χ^2(3:1)	*P*
	Total	Green	Albino		
Pei'ai 64S/*tcd3*	381	298	83	2.10＜3.84	0.15＞0.05

$\chi^2_{0.05}$=3.84

Chloroplast development at low temperature requires the pseudouridine synthase gene *TCD3* in rice

Dongzhi Lin· Rongrong Kong· Lu Chen· Yulu Wang· Lanlan Wu· Jianlong Xu·Zhongze Piao· Gangseob Lee· Yanjun Dong

Table S2 The PCR-based molecular markers designed for fine mapping

No.	Markers	Forward sequence (5' to 3')	Reverse sequence (5' to 3')
1	MM0541	TTTGTTTTCCCTTGGCTTTG	GCGCTAGGAGTTGGAACGTA
2	RM14407	GCAGAATATCCTAGCGTCAATGG	TCTTGTAACCTGAAACGGATGG
3	RM4683	TTGAGCATCAGGGTACTTTCTCC	GGAGTATTGTGCTAGTCACGTTTGG
4	ID2700	TCTCGTCTTGTGCCGTTAGC	CTTCGCTTTGGTTTTGGGTC
5	ID2738	GCTTGTGTGGACTTCAGGCG	GAACTGTTCCTAGCAGCGCA
6	ID2917	TGTTTGGGCAGCGAGTAAGTG	ATTGGAATAGGGAAAGTGAGCA
7	ID3045	TGAAAGAGAACAAGCCCGAAG	AACCCCGTTGGAAGAACCTAC

Chloroplast development at low temperature requires the pseudouridine synthase gene *TCD3* in rice

Dongzhi Lin· Rongrong Kong· Lu Chen· Yulu Wang· Lanlan Wu· Jianlong Xu·Zhongze Piao· Gangseob Lee· Yanjun Dong

Table S3 Markers designed for realtime RT-qPCR and gene function

Genes	Primer sequences (5' to 3')	Gene functions
CAO1	GATCCATACCCGATCGACAT<br>CGAGAGACATCCGGTAGAGC	Encoding chlorophyllide a oxygenase1(Oster *et al.*, 2000)
HEMA	CGCTATTTCTGATGCTATGGGT<br>TCTTGGGTGATGATTGTTTGG	Encoding glutamyl tRNA reductase (McCormac *et al.*, 2001)
PORA	TGTACTGGAGCTGGAACAACAA<br>GAGCACAGCAAAATCCTAGACG	Encoding NADPH-dependent protochlorophyllide oxidoreductase(Sperling *et al.*, 1997)
YGL1	CAGTCTCCAATGGCCACCT<br>TGCTTTCATCAGTGGCTGGT	Encoding a Chl synthetase (Wu *et al*., 2007)
cab1R	AGATGGGTTTAGTGCGACGAG<br>TTTGGGATCGAGGGAGTATTT	Encoding light-harvesting Chl a/b binding protein 1(Luan and Bogorad, 1989)
LHCPII	GAAGAAGATCAAGAACGGCC<br>TTGCCGGGGACGAAGTTGGT	Encoding light-harvesting complex protein in PS II (Murray and Kohorn 1991)
psaA	GCGAGCAAATAAAACACCTTTC<br>GTACCAGCTTAACGTGGGGAG	encoding the P700 chlorophyll a apoprotein of PS I (Steiner *et al.,* 2009)
psbA	CCCTCATTAGCAGATTCGTTTT<br>ATGATTGTATTCCAGGCAGAGC	Encoding the D1 protein of PS II (Steiner *et al*., 2009)
rbcL	CTTGGCAGCATTCCGAGTAA<br>ACAACGGGCTCGATGTGATA	Encoding the large subunit of Rubisco(Barkan *et al.*, 1993)
rbcS	TCCGCTGAGTTTTGGCTATTTGGACTTG<br>AGCCCTGGAAGG	the small subunit of Rubisco(Wanner *et al.*,1991)
atpA	GACAGACTGGCAAAACAGCA<br>CCGTTCGCGGTACATAAAAT	Encoding a protein of ATP synthase (Drapier *et al.*,1992)
FtsZ	AAAGGACATAACCTTGCAAG<br>AGTTTTCCTATTGAACCGTG	Encoding a protein of chloroplast division process(Stanislav *et al*., 2001)
OsPOLP	ACCGGTGCTTTCAGGCTTGG<br>GCTGACTGATAATCACACG	Encoding one plastid DNA polymerase(Takeuchi *et al.*, 2007)
OsRpoTp	AAGCAGACAGTGATGACATC<br>ATCACATGCATGCACCCAAA	Encoding RNA polymerase subunit of PEP (Hiratsuka *et al.*, 1989)
Osv4	ACATGGTCGCCGTCTTCCGC<br>GCTTGCCAGCACTGTCACGA	Encoding chloroplast development related protein(Gong *et al.*, 2014)
rpoB	TTTGGTTTCGATGTGCA<br>TATGGTCTAATTCCGAGCGGT	Encoding RNA polymerase β subunits of PEP(Little *et al.*, 1988)
rpoC	ATTAGACGCATGCAATTGGC<br>CAATGGGTCTTAATTCGGGA	Encoding RNA polymerase β' subunits(Little *et al.*,1988)
rps7	GCCAAAATCCATTCCAATTC<br>GGAGATGTACACGAGGAGATTG	Encoding the small subunits ribosomal protein S7 (Joshua J *et al*.,2009)
rps20	CACGCTCTTCTCCCTCTCCT'<br>GTAGGAGGCGGACAGGCG	Encoding the small subunits ribosomal protein S20 (Nieminen et al .2011, Gong *et al.*, 2013)
S4	AAAATACGCCGTCTGGGAGC<br>TGTCAGACCATAATGAAAACGC	Encoding the small subunits ribosomal protein S4(Heilek *et al.*, 1995)
V1	TCAGAACGAGAAGGATTCAGCA<br>GGCAACAGCCACTAAAATTTCT	Encoding a chloroplast localized protein NUS1(Kusumi et al.1997)
V2	GAGGAGTTCCTCACGATGAT<br>AGCATCAATGATAGACTCC	Encoding plastid/mitochondrial guanylate kinase (pt/mtGK) (Sugimoto et al.2007)
V3	GTTAGATGCTTCACTACACAG<br>GTACCATTGCCAACATGGCAAC	Encoding the large subunits of ribonucleotide reductase (RNRL)(Hiratsuka et al.1989)
16SrRNA	CCGTTGGTGTTCTTTCCGAT<br>TTCAAGTCCGCCGTCAAATC	Encoding chloroplast ribosomes small subunits of 16S components (Little et al.1988)
23SrRNA	TGTGGGCGTTAGAGCATTGAG<br>CACTTGGCTACCCAGCGTTTA	Encoding chloroplast ribosomes large subunits of 23S components(Little et al.1988)
TCD3	TATCAAGGAGCACGAACCGC<br>GCAGACAGAACCATTGACGG	Encoding pseudouridine uridine synthase protein
OsActin	AGGAAGGCTGGAAGAGGACC<br>CGGGAAATTGTGAGGGACAT	As the internal control (Wu et al.2007)
